# Detection of Norovirus genogroup I and II by multiplex real-time RT- PCR using a 3'-minor groove binder-DNA probe

**DOI:** 10.1186/1471-2334-6-69

**Published:** 2006-04-10

**Authors:** Marina Hoehne, Eckart Schreier

**Affiliations:** 1 Robert Koch-Institute, Nordufer 20, 13353 Berlin, Germany

## Abstract

**Background:**

Due to an increasing number of norovirus infections in the last years rapid, specific, and sensitive diagnostic tools are needed. Reverse transcriptase-polymerase chain reactions (RT-PCR) have become the methods of choice. To minimize the working time and the risk of carryover contamination during the multi-step procedure of PCR the multiplex real-time RT-PCR for the simultaneous detection of genogroup I (GI) and II (GII) offers advantages for the handling of large amounts of clinical specimens.

**Methods:**

We have developed and evaluated a multiplex one-tube RT-PCR using a combination of optimized GI and GII specific primers located in the junction between ORF1 and ORF2 of the norovirus genome. For the detection of GI samples, a 3'- minor groove binder-DNA probe (GI-MGB-probe) were designed and used for the multiplex real-time PCR.

**Results:**

Comparable results to those of our in-house nested PCR and monoplex real-time-PCR were only obtained using the GI specific MGB-probe. The MGB-probe forms extremely stable duplexes with single-stranded DNA targets, which enabled us to design a shorter probe (length 15 nucleotides) hybridizing to a more conserved part of the GI sequences. 97 % of 100 previously norovirus positive specimens (tested by nested PCR and/or monoplex real-time PCR) were detected by the multiplex real-time PCR. A broad dynamic range from 2 × 10^1 to 2 × 10^7 genomic equivalents per assay using plasmid DNA standards for GI and GII were obtained and viral loads between 2.5 × 10^2 and 2 × 10^12 copies per ml stool suspension were detected.

**Conclusion:**

The one-tube multiplex RT real-time PCR using a minor groove binder -DNA probe for GI is a fast, specific, sensitive and cost-effective tool for the detection of norovirus infections in both mass outbreaks and sporadic cases and may have also applications in food and environmental testing.

## Background

Norovirus (NV), one genus of the family of *Caliciviridae*, is one of the most commonly reported etiological agents of non-bacterial gastroenteritis in human world-wide. In Germany, increasing numbers of NV cases have been reported to the public health authorities between 2001 (9,223 reports) and 2004 (64,893 reports) [1,2]. Especially, in the winter seasons 2002/2003 and 2004/2005 (October to April) a substantial increase of norovirus outbreaks have been detected in Germany as well in Western Europe [3,4]. Hospitals, residential facilities/nursing homes and schools/nurseries were most frequently affected. To prevent further spreading of the causative agent during a mass outbreak especially in semi closed communities, such as hospitals and nursing homes, an immediate application of hygiene measures as well as rapid and sensitive diagnostics are needed. The increasing knowledge of the molecular properties of caliciviruses led to the development of different assays for the detection of norovirus RNA and viral antigen (reviewed in [5]). Since enzyme immunoassays were found to be insufficient sensitive and/or insufficient specific so far [5-9] RT-PCR assays have become the methods of choice [10-13]. To minimize the working time and the possibility of carryover contamination during the multi-step procedure of RT-PCR, increasing attention has been paid to the detection by fluorogenic real-time PCR [14-18]. Due to the high sequence diversity of human NV which is classified into 3 genogroups (GI, GII, and GIV) containing at least 7 different genotypes in GI and 12 genotypes in GII [19,20] the optimization of primers and probes are crucial. Recently, we have reported the application of a one-tube real-time PCR using two primer/probe sets for the detection of NV GI and GII sequences in separate assays (monoplex real-time PCR) [21,22]. Using the genogroup specific oligonucleotide probes used in this monoplex GI and GII real-time PCR the combination in a multiplex PCR resulted in ineffective detection. Recently, the implementation of a minor groove binding protein (MGB) and nonfluorescent quencher (NFQ) significantly improved the chemistry of the real-time PCR [23]. In this study, we applied a GI specific MGB-probe which enables us to develop a broadly reactive and sensitive multiplex real-time RT- PCR for the simultaneous detection of both genogroups using a TaqMan^® ^7700. Due to the utilization of two different fluorophores (FAM and VIC) for the probes a differentiation between GI and GII genotypes as well as the detection of double infection with GI and GII genotypes is possible.

## Methods

### Clinical specimens

Altogether, 100 NV positive stool specimens and 40 NV negative specimens were used to evaluate our multiplex real-time RT-PCR. The stool specimens were obtained between January 2004 to June 2005 from 39 norovirus derived outbreaks and 31 sporadic cases in various geographical areas in Germany as well as from 40 healthy individuals without any sign of gastroenteritis. All samples were previously tested by our in house RT/nested PCR [11] and/or the monoplex real-time PCR described earlier.

### Sample processing

Viral RNA was extracted from stool specimens using QIAamp™ Viral RNA Mini Kit (QIAGEN, Hilden, Germany) and subjected to our ORF1 in house RT/nested PCR and to the monoplex real-time PCR. For genotyping the ORF1 amplicons were directly sequenced using the BigDye terminator cycle sequencing kit and an ABI Prism 3100 Genetic Analyzer (Applied Biosystems, Foster City, USA). Sequences were aligned to prototype sequences drawn from GenBank using CLUSTAL W version 1.6 and phylogenetic trees were produced using the neighbour joining and DNADIST program of the Phylogeny Interference Package (PHYLIP) version 3.57c [24].

### One tube multiplex real-time RT-PCR

Sequences of oligonucleotide primers for the monoplex real-time PCR of NV GI and GII and the GII specific probe have been described earlier. The optimized primer and probe sequences used for the multiplex real-time RT-PCR are listed in table 1. The sense primer for GII (NV107a) was supplemented with an oligonucleotide primer containing inosin at the more variable nucleotide positions (NV107c). All primers were obtained from BioTez (Berlin, Germany). The MGB-GI probe (Applied Biosystems UK) was labelled at 5'-term with the fluorophor VIC and at the 3'-term with MGB/non fluorescent quencher (NFQ) dabcyl and the GII probe (TIB MOLBIOL, Berlin, Germany) was labelled at 5'-term with 6-FAM and 3' with NFQ. The single-tube multiplex real time RT-PCR was carried out in 0.2 ml tubes (96-well PCR plates, Thermo-Fast 96, Abigene, Surrey, UK) using a TaqMan^® ^7700. The reaction was performed in 12 μl volumes using the QuantiTect Probe RT-PCR Kit from QIAGEN (Hilden, Germany) containing 1x Quantitect Probe RT-PCR Master Mix (including HotStar Taq DNA polymerase, Tris-HCl, KCl, (NH_4_)_2_SO_4_, 4 mM MgCl_2, _dATP, dCTP, dGTP, dTTP, dUTP and the internal reference dye ROX), 0.2 μM of each primer as described in table 1; 80 nM GI TM9 MGB probe; 160 nM GII TM3A probe and 0.2 μl of QuantiTect Probe RT Mix containing Omniscript and Sensiscript reverse transcriptases. Two microliters of sample RNA preparation or standard DNA were added to each reaction. Thermal cycling for the TaqMan 7700 was performed as follows: 30 min at 50°C for reverse transcription, 15 min at 95°C for heat inactivation of the reverse transcriptases and the initial activation of the HotStar polymerase, 45 cycles of 20 seconds at 94°C and 30 seconds at 60°C. The fluorescence data were collected at the end of the 60°C step. To generate a standard curve 10-fold serial dilution of plasmid-DNA containing the appropriate GI or GII sequences were used as described earlier.

**Table 1:** Primers and probes used for one tube multiplex real-time RT-PCR

Genogroup	Primer	Sequence (5' – 3')	Location*
G I	NV192 (s)	5'-GCYATGTTCCGCTGGATGC	5282–5300
	NV193 (as)	5'-CGTCCTTAGACGCCATCATCA	5379–5359
	TM9-MGB probe	5'-VIC-TGGACAGGAGATCGC-MGB-NFQ	5345–5359
G II	NV107a (s)	5'-AGCCAATGTTCAGATGGATG	5007–5026
	NV107c (s)	5'-AICCIATGTTYAGITGGATG	5007–5026
	NV119 (as)	5'-TCGACGCCATCTTCATTCAC	5100–5081
	TM3A probe	5'-6'FAM-TGGGAGGGCGATCGCAATCTGGC-NFQ	5048–5070

*Genome location of primers and probes are based for GI on the sequence of Norwalk/68/US [GenBank: M87661] and for GII on the sequence of Lordsdale/93/UK [GenBank: X86557]; Y= C/T; I= inosin; MGB= minor groove binder; NFQ= non fluorescent quencher

## Results

All results of the multiplex real-time RT-PCR were compared to the results achieved by our in-house nested RT-PCR and our monoplex real-time PCR. Sequence analysis revealed that the panel of 100 NV positive specimens consisted of 4 different GI and 8 different GII genotypes/subtypes including naturally occurring recombinants (polymerase region: GIIb but capsid region: GII.3 [25,26]) as well as 2 new GII genotypes which were not classified up to now (table 2). As increasingly numbers of new NV sequences can be observed in GenBank updates of primers and probes were necessary. To ensure the detection of a broad range of NV genotypes using the multiplex TaqMan RT-PCR we used the GII sense primer NV 107 as a mixture of the original described primer NV107a and an inosin-containing primer NV107c located at the same position. All final primer sequences as well as the design of the GI-MGB-probe and the GII-probe are shown in table 1.

**Table 2:** Detection of norovirus of different genotypes by multiplex real-time-PCR. Comparison to numbers of NV positive specimens as tested by nested PCR/monoplex real-time PCR

Genotype*	**Prototype strain**	**GenBank no.**	**Detection by multiplex TaqMan (no. pos./no. tested**
I.1	Norwalk//1968/USA	M87661	1/1
I.2	Southampton/1991/UK	L07418	2/2
I.3	Desert Shield395/1990/SA	U04469	7/7
I.6	Sindlesham/1995/UK	AJ277615	2/2
II.2	Melksham/1989/UK	X81879	2/3
II.3**	Arg320/1995/AR	AF190817	4/4
II.4	Grimsby-like 2002	AY485642	3/3
II.4	Grimsby-like 2004	AY883096	60/62
II.7	Leeds/1990/UK	AJ277608	9/9
II.10	Erfurt/546/2000/GE	AF427118	1/1
II.unclassified	distantly related to	AY673935	2/2
II.unclassified	distantly related to	AB084786	1/1
II. not determ.			3/3

summary			97/100 (97 %)

NV negative			0/40 (100 %)

* Genotype classification according to [19]

** Genotype classification in the polymerase region: GIIb/Hilversum, in the capsid region GII.3 [26]

**Figure 1:**
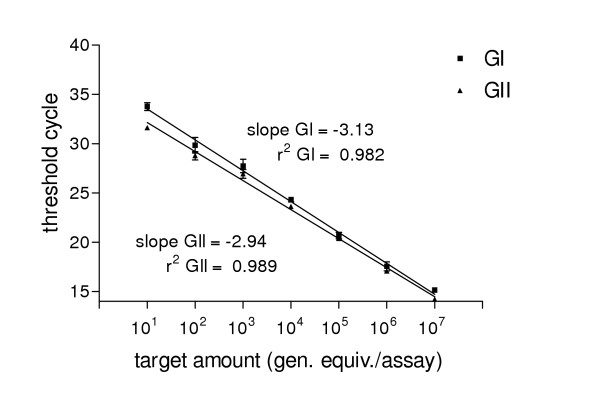
Standard curves of norovirus multiplex real-time PCR. 10-fold serial dilution of plasmid DNA (10^1 ^to 10^7 ^genomic equivalents per assay) of GI and GII were plotted versus C_t _value. Each dot represents the average of three reactions. Error bars indicate standard deviations.

**Figure 2:**
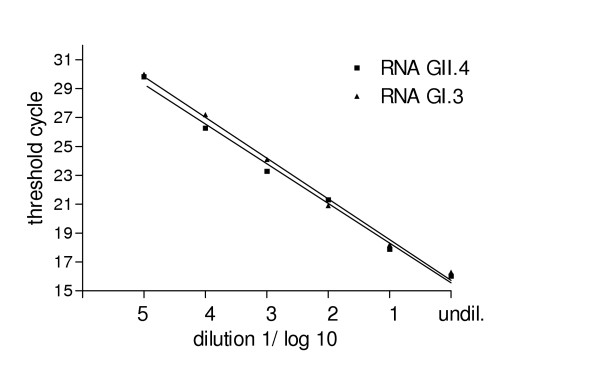
Ten-fold serial dilution of viral RNA. RNA obtained from two patients infected with norovirus GI.3 and GII.4 (Grimsby 2004) were diluted and amplified by the multiplex real-time RT-PCR. The undiluted RNA's contained 10^6 ^genomic equivalents per assay.

The dynamic range of our multiplex real-time RT-PCR was determined using a 10-fold serial dilution of a GI and a GII plasmid DNA standard containing the appropriate gene fragment as described earlier. The standard curves for GI and GII showed linearity between 2 × 10^7 ^and 2 × 10^1 ^genome equivalents per assay with a slope of -3.13 and -2.94 for GI and GII, respectively (fig. 1). The coefficients of correlation of r^2^= 0.98 and 0.99 indicate a strong linear relationship. No cross-reactivity between GI and GII using the two different dye-layers of FAM and VIC were observed. Using 10-fold serial dilutions of viral RNA prepared from a GI.3 and a GII.4 positive stool specimen containing about 10^6 ^copies/assay the multiplex real-time RT-PCR was able to detect RNA dilutions within 5 logs (fig. 2). Thus, about 10–20 genomic equivalents per multiplex TaqMan RT-PCR assay could be detected which is comparable to the detection limit of the monoplex real-time PCR.

**Figure 3:**
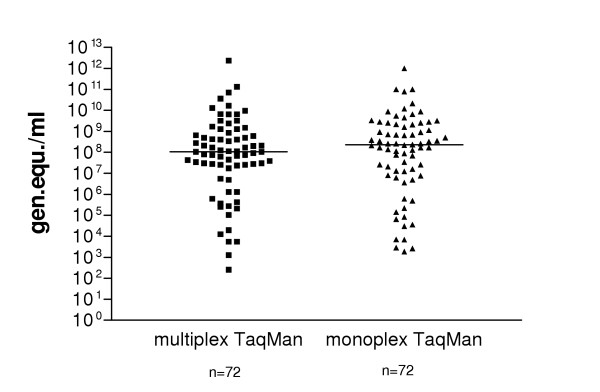
Comparison of viral loads determined by multiplex and monoplex real-time PCR. Viral RNA's from 72 stool samples were used. Virus loads between 10^3 ^and 10^12 ^(median 2.27 × 10^8^) and between 10^2 ^and 10^12 ^genomic equivalents (median 1.05 × 10^8^) were detected by monoplex and multiplex real-time RT-PCR, respectively.

Overall, 97 out of the 100 nested PCR and/or monoplex real-time PCR positive specimens were positive in the multiplex assay (sensitivity: 97 %). All 40 NV negative specimens were also negative in the multiplex-TaqMan PCR. Samples of all 12 different NV genotypes/subtypes tested (4 GI and 8 GII genotypes) were detectable (table 2). To evaluate the quantities detected by the multiplex TaqMan PCR we compared the viral loads to those detected by the monoplex TaqMan-PCR using 72 different stool samples (fig. 3). The median of viral load of specimens detected by the multiplex and the monoplex TaqMan-PCR, respectively, were found to be 1.05 × 10^8 ^and 2.27 × 10^8 ^genomic equivalents per ml. A P value of 0.6 as determined by the paired t-test showed that the means were not significant different (p > 0.05). Using 4 different stool specimens (two GI.3 and two GII.4) in 3 individual assays the coefficients of variation of Ct values were between 0.25 % and 4.02 %.

Three mixtures of known copy numbers of GI and GII plasmid DNA containing each 10^6^, 10^4^, or 10^2 ^genomic equivalents as well as four viral RNA mixtures (each 10^6^, 10^5^, 10^4^, and 10^2 ^genom. equ.) were amplified by the multiplex real-time assay. As shown in table 3, GI and GII could be detected in all mixtures with a good correlation to the expected values between 10^6 ^and 10^4 ^genomic equivalents. Also in the mixtures containing the highest dilution (10^2 ^copies) both genotypes could be detected but with more variation from the expected value.

**Table 3:** Detection and quantitation of GI/GII mixtures using the multiplex real-time RT-PCR

	Expected quantity (copies/assay)		Detected quantity (copies/assay)	
	GI	GII	GI	GII
plasmid DNA	10^6^	10^6^	1.2 × 10^6^	1.4 × 10^6^
	10^4^	10^4^	2.2 × 10^4^	1.8 × 10^4^
	10^2^	10^2^	2.6 × 10^1^	5.1 × 10^0^
				
viral RNA	10^6^	10^6^	3.3 × 10^6^	4.7 × 10^6^
	10^5^	10^5^	4.0 × 10^5^	3.2 × 10^5^
	10^4^	10^4^	4.4 × 10^4^	2.7 × 10^4^
	10^2^	10^2^	6.0 × 10^3^	5.0 × 10^0^

Known copy numbers of GI and GII plasmid DNA and GI and GII viral RNA were mixed and analyzed by the multiplex real-time RT-PCR

## Discussion

For the diagnostic of increasing numbers of stool samples from single cases of viral gastroenteritis infections and especially in mass outbreaks occurring in the winter seasons a fast, sensitive and specific assay is required. Due to the genetic diversity of human noroviruses the selection of a highly conserved genome region is very important for the design of universal primers and probes for all genotypes. Multiple sequence analysis showed that the highest nucleotide homology is located in the ORF1 -ORF2 junction [14]. Nevertheless, differences between GI and GII sequences are considerable in this region so that different primers/probes sets including two different or wobbled GI probes or SYBR Green I detection instead of specific probes had to be used by several author's [14,16,17,22,27]. To simplify the real-time PCR and to reduce the costs, we developed a multiplex one-tube real-time RT-PCR exhibiting a high sensitivity and specificity while being broadly reactive for both genogroups. In the method described here the performance of the GI probe was increased by the use of the MGB probe technology. TaqMan-MGB probes are more stable and show an improved signal-to noise ratio due to the use of non-fluorescent quencher (NFQ) instead of the fluorescent quencher TAMRA. The MGB stabilizes the hybridization of the probe leading to an increased melting temperature even for very short oligonucleotides of 13 – 15 nucleotide length. For that reason, the length of our GI specific TaqMan probe could be optimized to fit to a shorter, more conserved region of all GI genotypes known. Thus, in the contrary to Kageyama et al. [14] and Pang et al. [27] only one GI specific probe is needed for our multiplex real-time PCR and the dynamic range had been extended by up to two orders of magnitude for the GI standard curve. Although, the GII.4 norovirus subtypes (Grimsby, Grimsby 2002 and Grimsby 2004) [28-30] have been the predominant strains since 2002 [29,31] different genotypes are co-circulating in Europe and worldwide and new strains have been detected and characterized [20]. The genotypes tested by our multiplex real-time PCR represent the majority of noroviruses circulating in Germany in the last two years. Using the primers/probes described here for the multiplex real-time PCR, we were able to detect at least all 12 different genotypes/subtypes tested including 4 specimens related to the naturally occurring recombinants like Arg320/1995/AR [AF190817] and Bad Berleburg477/2001/DE [AF409067] [25,26]. The three negative tested samples (two GII.4 and one GII.2) may related to a very low copy number probably due to degradation of viral RNA during several freeze/thawing cycles during the long-term storage. Furthermore, two so far unclassified GII genotypes have been detected, one strain distantly related to the sequence of the polymerase fragment of GenBank: AB084786 and two samples distantly related to GenBank AY673935. Although, our multiplex real-time PCR was able to detect two new genotypes a further update of primer/probe sequences should be considered if new, aberrant norovirus sequences are available at EMBL or NCBI databases. Using two different fluorophores for the genogroup-specific probes the multiplex RT- real-time PCR permits the allocation to genogroup I or II or even the detection of mixtures of genogroups in one sample. This might be of special interest for the norovirus detection in wastewater or in contaminated sea food, such as oysters or shellfish.

## Conclusion

We have developed a broadly reactive, fast and sensitive one-tube RT/real-time PCR for the simultaneous detection and quantitation of human norovirus genogroup I and II. Due to the application of a MGB-probe the specificity and sensitivity of the multiplex real-time PCR is comparable to that of the monoplex real-time PCR but saving time and costs. Thus, the multiplex real-time PCR might be useful for the norovirus detection in mass outbreaks and sporadic cases of gastroenteritis as well as in contaminated seafood and waste water.

## Competing interests

The author(s) declare that they have no competing interests.

## Authors' contributions

MH designed the study, carried out the assay development, data analysis, and drafted the manuscript. ES participated in the design of the study, provided expert input, and critically reviewed the manuscript. Both authors have read and approved the manuscript.

## Pre-publication history

The pre-publication history for this paper can be accessed here:


